# Support exchange between siblings in later life: the role of “core-kinlessness” and geographic proximity

**DOI:** 10.1093/geronb/gbag108

**Published:** 2026-06-20

**Authors:** Alyona Artamonova, Clara H Mulder, Mirkka Danielsbacka, Antti O Tanskanen

**Affiliations:** INVEST Research Flagship Centre, University of Turku, Turku, Finland; Population Research Institute, Väestöliitto, Helsinki, Finland; Faculty of Spatial Sciences, University of Groningen, Groningen, The Netherlands; INVEST Research Flagship Centre, University of Turku, Turku, Finland; Population Research Institute, Väestöliitto, Helsinki, Finland; Population Research Institute, Väestöliitto, Helsinki, Finland; Department of Social Research, University of Turku, Turku, Finland; (Social Sciences Section)

**Keywords:** Intragenerational relationships, Personal care, Practical help, Geographic closeness, Kinlessness

## Abstract

**Objectives:**

As people have fewer children and remain single, siblings may become more active in family networks of older adults who lack a partner, children, or both—referred to as “core-kinless.” This study examines the association between the absence of children and/or a partner and the exchange of support between siblings and how intragenerational geographic proximity shapes these associations, addressing both giving and receiving practical help and giving personal care.

**Methods:**

Data from the Generational Transmissions in Finland project (adults aged 78–74 in 2018 and their siblings), linked with register data, were analyzed using multilevel ordinal logistic regressions to examine the frequency of giving and receiving practical help, and logistic regressions providing personal care.

**Results:**

Siblings were found to be active supporters in later life. Partnerless older adults—particularly never married—were more likely to receive practical help from siblings than those with partners. Childlessness alone did not increase sibling support, but core-kinless older adults received the highest levels of help and provided more assistance and personal care to siblings, even across long distances. Geographic proximity strengthened support exchanges, but core-kinlessness remained a strong predictor of support exchange regardless of distance. Finally, a 10 km threshold seems adequate for approximating help and care exchanges between siblings.

**Discussion:**

Findings highlight siblings’ role as a compensatory source of support in later life, especially if they are core-kinless. Future research and policy should broaden caregiving definitions to include siblings and recognize core-kinless older adults as an underappreciated resource of help and care within family networks.

Population aging poses challenges to welfare states (Pani-Harreman et al., 2021), prompting increased attention to informal caregiving within families as a strategy to support older adults (Cattaneo et al., 2025; Pavolini & Ranci, 2008). While most research on family caregiving in later life has focused on spouses and children as core family members (see Connidis & Barnett, 2018), growing scholarly attention is being directed to individuals who lack a partner (referred to as “partnerless”) or children (“childless”). Older adults who lack both a partner and children are often referred to as “kinless” (e.g., Plick et al., 2021), although some researchers account for a more comprehensive array of absent kin to define kinless individuals, including siblings (e.g., Verdery et al., 2019) or parents and siblings (e.g., Margolis & Verdery, 2017). In this work, we use the term “core-kinless” to refer to older adults without a partner and children.

Older adults without a partner and children face elevated risks of loneliness and social isolation (Nicolaisen & Thorsen, 2014; Ong et al., 2016), institutionalization (Einiö, 2010; Korhonen et al., 2026; Syse et al., 2022), and premature mortality (Einiö et al., 2016; Patterson et al., 2020). The primary sources of support for these individuals may lie beyond the conventional nuclear family. Siblings, who are known to provide help and care when needed (Buchanan & Rotkirch, 2021; Eriksen & Gerstel, 2002), may be among the most overlooked potential helpers in this context.

Exploring the alternatives for providing help and care to older adults who are partnerless, childless, or core-kinless in Finland is timely for both policy and scientific reasons. Finland is a Nordic welfare state in which relatively extensive public services, including home help and other forms of publicly funded eldercare, are provided, and places primary responsibility for caregiving on the state (Kangas & Kvist, 2018). This context has also been described as “less familistic” (Leitner, 2003), and in such a context, public provision may reduce or substitute for family-based care, potentially limiting the need for kin support. However, rapid population aging in Finland is creating fiscal pressures that may constrain publicly funded care services. People aged 65 and over account for 24% of the adult population (Rotkirch, 2021), and their number is expected to double by 2040 (Marois et al., 2022). In practice, the gradual retrenchment of formal support may lead to a growing expectation that families will assume greater responsibility for meeting the care needs of older individuals (Rostgaard et al., 2022). For older adults without core kin, siblings may represent the closest available family members and thus the most likely source of informal support. This study therefore examines whether siblings assume a compensatory role in meeting care needs, even within a welfare state context where formal services remain widely available.

Although sibling relationships are regarded as secondary compared with spouses and children in adulthood (Rossi & Rossi, 1990), they represent one of the most enduring social ties over the life course (Gold, 1987; White, 2001). Similar to other family relationships, they are characterized by family solidarity (i.e., feelings of mutual connectedness expressed in behavioral terms) (Knijn & Liefbroer, 2006). Relationships are tested on their solidarity in the face of adversity (Voorpostel, 2007). Research shows that family members, including siblings, indeed, not only feel obligated to support each other when needs appear (Rossi & Rossi, 1990) but in practice provide each other with help, suggesting that sibling solidarity remains strong in contemporary societies (Tanskanen & Rotkirch, 2019). In the absence of the core ties (i.e., among those who never marry or never have children), siblings should be important, as suggested by the hierarchical-compensatory model (Cantor, 1991) and the substitution hypothesis (Shanas, 1979). These models argue that individuals follow a normative order of preference for support, turning to other network members, including siblings, when preferred sources such as partners and children are unavailable.

Sibling relationships may also regain prominence after the loss of core kin, as the functional specificity of relationships model assumes (Connidis, 1994; Simons, 1983–1984). According to this model, family relationships are flexible and negotiated; they can serve distinct support functions, and their activation depends on contextual needs and available alternatives. In line with this model, there might be differences in how relationships with siblings are negotiated between those who have never married and those who lost a partner via divorce or death. More specifically, the never-married could establish supportive ties and close geographic proximity with their siblings in earlier life course stages, while those who were married but lost their partners might have a different system of support (Connidis, 1994) represented (e.g., by their former partner’s families). Importantly, widowed older adults can find themselves in a more advantageous position in terms of access to social networks beyond siblings relative to the divorced (Lopata, 2017) as divorce is known to have deleterious effects on social networks (Terhell et al., 2004).

Older adults without a partner and/or children may become not only recipients but also providers of help and care for their siblings. According to the convoy model of social relations (Kahn & Antonucci, 1980), when a partner and children are absent, older adults may redefine their caregiving identity within the available social network and redirect their caregiving toward other close kin. We therefore focus on both giving and receiving sibling support in this study.

Geographic proximity between family members is a key determinant of the exchange of support between them (Eriksen & Gerstel, 2002; Knijn & Liefbroer, 2006). The “distance decay” in support, however, may differ between configurations of family networks (Mulder & Van der Meer, 2009). Individuals who feel greater responsibility for someone’s well-being (e.g., those whose family members lack alternative caregivers) may be more willing to travel longer distances to provide support. Therefore, individuals might experience greater pressure to support their siblings if those siblings do not have a spouse and children, irrespective of the distance between them. Importantly, support can range from practical help with housekeeping or paperwork, which tends to be provided on occasion, when the provider has the opportunity, to regular physical care that addresses specific needs (Brandt et al., 2009). Care requires close geographical proximity between the recipient and provider for adequate provision relative to practical help (Brandt et al., 2009; Hicks et al., 2018). We, therefore, explore the relationship between support exchange, lacking core kin, and intragenerational geographic proximity separately for practical help and personal care.

Distance might also play a part in the likelihood of calling upon siblings for help. Older adults may be less inclined to acknowledge their needs to siblings who live far away because they do not want to burden those who cannot easily provide support (Avioli, 1989). However, in the absence of core family members, siblings might be called upon for help regardless of distance. Finally, older adults without children and partners might be more willing and able to provide help and care to siblings irrespective of distance, compared with older adults with these core family members who likely face competing family obligations.

Consistent with theoretical reasoning from social support models, previous studies have shown that older adults without partners or children are more likely to rely on siblings for support (Choi, 1994; Fihel et al., 2021). However, empirical research on the distances at which siblings provide support to core-kinless older adults remains limited. Register data show that childless and unpartnered older adults are more likely to move closer to their siblings (Artamonova & Gillespie, 2022) and to live within a 10-km radius of their neighborhood (Artamonova & Gillespie, 2023), but these records do not provide information on actual support exchange. It is therefore unclear whether this geographic proximity is accompanied by an increase in practical help that core-kinless receive from siblings or care they provide to them. Furthermore, it remains unknown whether core-kinless older adults receive and give more help even if they do not live close to their siblings. In light of the above-mentioned theoretical insights and empirical gaps, we examine (a) *how the absence of children and/or a partner is associated with the exchange of practical help between siblings and giving personal care to siblings in later life* and (b) *how intragenerational geographic proximity shapes these associations*.

We derive four hypotheses regarding the relationship between not having core family members, intragenerational geographic proximity, and support between siblings in later life:

H1: Partnerless individuals will give and receive practical help from siblings more frequently and will be more likely to give personal care to siblings than those who have a partner;

H2: Childless individuals will give and receive practical help from siblings more frequently and will be more likely to give personal care to siblings than those who have children;

H3: Core-kinless individuals will give and receive practical help from siblings more frequently and will be more likely to give personal care to siblings than those who have both a partner and children;

H4: Individuals will give and receive practical help from siblings more frequently and will be more likely to give personal care to siblings who live nearby than those who live far away. However, we expect this association with distance to be weaker for core-kinless index persons than for those with core kin.

Several additional sources of heterogeneity can be expected to influence both the propensity for help and care exchange with siblings and the role that proximity plays in this exchange. On the one hand, poor health and older age might correspond to individuals’ need for care and practical help (Kalmijn & Saraceno, 2008). On the other hand, health and age relate to the potential to provide support to family members (Eriksen & Gerstel, 2002). Gender configuration is linked to involvement in sibling relationships with sisters being the closest and most involved sibling dyads, sisters–brothers in the middle, and brothers the most distant (Gold, 1987; Stocker et al., 2020; Tanskanen & Rotkirch, 2019). Full biological siblings tend to be closer emotionally and have more contact with each other than half siblings (Gilligan et al., 2020; Tanskanen et al., 2021) and, therefore, might be more involved in support exchange. Individuals with greater family incomes are more likely to provide support to siblings with fewer resources (Eriksen & Gerstel, 2002). We also include the vital status of parents, as sibling interaction tends to intensify after parental death (Kalmijn & Leopold, 2019). Urban residents live closer to their family members than inhabitants of sparsely populated regions do (Malmberg & Pettersson, 2007). As Swedish speakers in Finland have higher levels of social capital (Hyyppa, 2010), lower divorce rates (Saarela & Finnäs, 2018), and more children (Rotkirch et al., 2018) than Finnish speakers, differences in sibling roles between these two linguistic groups can be expected. Finally, since the distribution of time and energy among more siblings may make solidarity toward any given sibling less likely (Dykstra & Knipscheer, 1995; Eriksen & Gerstel, 2002), we consider the total number of siblings.

## Research design and methods

### Data

To answer our research questions, the present study utilizes survey data from the Generational Transmissions in Finland (Gentrans) project linked to register data. The Gentrans data incorporate information on the Finnish baby boomer generation born between 1945 and 1950. The data were collected by Statistics Finland in 2018–2019, and comprised a nationally representative sample of 2,663 older adults aged 68–74 years. The surveys were self-administered paper questionnaires that were mailed to respondents and returned via mail. Respondents were also given the option to access and submit the questionnaire online. The response rate was 66.4%, of which 8.3% were online submissions. As the survey did not include information on the respondents’ place of residence, we extracted an urbanity measure from population registers.

We denote the survey respondent as the index person in a dyad. The criterion for inclusion in the sample was that the index person had at least one living sibling. We excluded employed respondents who were still bound to a work location, as we wanted to focus on a relatively heterogeneous population in a specific life stage. The index persons could answer about up to four siblings (starting from the oldest). The selection of siblings for whom information on support exchange was collected implies that dyads from large families are somewhat underrepresented. The analyses are based on information about 4,040 dyads nested within 1,699 older adults in the models for the frequency of receiving practical help; 4,044 dyads within 1,698 older adults in the models for the frequency of giving practical help; 4,038 dyads within 1,694 older adults in the models for the likelihood of providing personal care. The numbers vary slightly due to missing values in respondents’ answers about intragenerational exchanges.

### Dependent variables

In Gentrans, respondents were asked whether they (a) received practical help from siblings and (b) gave practical help to siblings (e.g., with housework, yard work, renovation, grocery shopping, or using technology) in the past 12 months before the interview. Those who answered “yes” were asked to report the frequency of receiving and giving such help. For the analysis, these responses were combined into two ordinal outcome variables: (a) *frequency of receiving* and (b) *frequency of giving practical help*, ranging from 0 to 2, where 0 = “No help”—the reference category (ref.), 1= “Less than once a month,” 2= “Once a month or more often.” Respondents were also asked whether they helped siblings with personal care (e.g., washing up, eating, or dressing) in the last 12 months and how frequently. Because *providing a sibling with personal care* was uncommon, we coded this variable as a binary outcome: giving personal care (1) or not giving it (0—ref.).

### Main independent variables

The main independent variables are the index persons’ partnership status, the presence and geographic proximity of children, the parenthood status of a sibling in a dyad, as well as the distance to this sibling. The partnership status of the index person included four categories: partnered (ref: married or cohabiting unmarried), unpartnered, separated, and widowed. We distinguished between those index persons who had at least one child within a 10-km radius (ref.), those with the closest child outside the 10-km radius, and those who had no children. For the measure of child’s closeness, we used the 10-km operationalization in line with previous studies on close intergenerational distance (Thomas & Dommermuth, 2020). A separate measure distinguished between index persons with both a partner and at least one child (ref.), those with neither, those with at least one child but no partner, and those who only had a partner. Our dataset did not include information on siblings’ partnership status, which prevented us from examining whether a sibling’s core-kinlessness affected the receipt of help and care from the index person. However, we did have a measure for whether siblings had children, categorized as: having at least one child (ref.) or not having children. Respondents were asked to report the distance to their siblings in km. Our recoded variable included six categories: 0–5 km, 6–10 km, 11–20 km (ref.), 21–50 km, 51–100 km, and 101+ km as used previously by Mulder and Van der Meer (2009).

### Control variables

We included several variables related to different dimensions of sibling relationships and needs for support at the levels of dyads and index persons. The *index person’s subjective health* included three categories: good (ref.), fair, and poor. *Siblings’ age* was categorized as ≤60 years, 61–65 years, 66–70 years (ref.), 71–75 years, 76–80 years, and 81+years. Respondents’ age was not included because of the small age range of 68–74 years (mean = 71, *SD* = 1.70).

We also included a measure of *emotional closeness between siblings* reported by the index person (continuous with a range from 1 to 5). We adjusted our models for the *gender composition of the sibling dyad*: index person was a sister–sibling was a sister (ref.), sister–brother, brother–sister, brother–brother. The *type of sibling ties* distinguished between full siblings (ref.), paternal half siblings, and maternal half siblings. The *joint subjective financial situation* variable was constructed based on the estimation of the index person and included four categories: both middle or higher income (ref.), index middle or higher and the sibling low, index low and the sibling middle or higher, both low income.

We controlled for a variable “*having at least one parent alive*” (no parents alive–ref.). We considered *index person’s regional urbanicity* (based on register data: rural–ref. or urban municipality). *Linguistic group affiliation* distinguished between Finnish (ref.) and Swedish speakers. Finally, we included the *total number of siblings* (continuous, range = 0–7+).

Most variables were taken from Gentrans; information about index persons’ marital status, language, and regional urbanicity was retrieved from registers. Descriptive statistics for all variables are presented in Supplementary Material (Table S1).

### Statistical analyses

Because the older adult–sibling dyads used as units of analyses are nested within index persons in all models, we employ multilevel ordinal and logistic regression models. The inclusion of the second level was statistically justified, as a likelihood-ratio test was statistically significant (*p* < .001).

We present three specifications for each dependent variable (nine models in total). The first specification includes index persons’ partnership status, the presence and geographic closeness of children, parenthood status of a sibling in a dyad, as well as distance to this sibling and control variables. The second specification contains a combination of the partnership and parenthood statuses instead of separate measures to distinguish core-kinless index persons. The third specification tests the interaction between the combination of the partnership and parenthood statuses of the index person and the geographic distance between siblings. We present the results in the form of coefficients and average predicted probabilities (APP).

In the main analysis, listwise deletion was used to handle missing data. Additionally, we ran our models based on the extended sample that includes individuals with missing values in control variables (with the category “unknown”; the results remained consistent with the main models based on a reduced sample). We also explored the robustness of our models using a distance categorization similar to the one employed in the Survey of Health, Aging and Retirement in Europe and alternative indicators of the index person’s health. Results for all sensitivity checks are presented in Supplementary Material.

## Results

### Descriptive results

In around 18% of dyads, index persons received practical help from siblings less than once a month and in 2.6% of dyads, they received it once a month or more often (Table 1). In 11.7% of dyads, index persons gave practical help to siblings less than once a month and in 2.4% of dyads, they gave it once a month or more often. Only in 3.5% of dyads, index persons provided their siblings with personal care.

**Table 1:** Bivariate results for the main explanatory variables: descriptive statistics (row percentages) of the dependent and independent variables.

Variables	Frequency of receiving help (*n* = 4,050)				Frequency of giving help (*n* = 4,044)				Giving care (*n* = 4,038)		
	No help	Once a month or less	More than once a month	χ^2^	No help	Once a month or less	More than once a month	χ^2^	No	Yes	χ^2^
Total	80.30	17.09	2.62	146.61 (Pr = 0.000)	85.86	11.72	2.42	48.19 (Pr = 0.000)	96.53	3.47	19.69 (Pr = 0.000)
**Index’s marital status**											
Unmarried/unpartnered	58.86	31.44	9.70		74.00	21.33	4.67		93.31	6.69	
Married	83.76	14.66	1.58		87.72	10.25	2.03		97.35	2.65	
Divorced/separated	79.64	17.50	2.87		85.95	11.63	2.42		94.99	5.01	
Widowed	73.46	22.52	4.02		81.67	14.82	3.50		95.92	4.08	
**Index’s parental status and child’s proximity**				75.91 (Pr = 0.000)				20.77 (Pr = 0.000)			4.53 (Pr = 0.104)
Index person is childless	69.48	23.22	7.30		80.11	16.89	3.00		95.27	4.73	
At least 1 child, >10 km	82.79	15.58	1.63		85.55	11.92	2.53		96.31	3.69	
At least 1 child, <11 km	81.18	16.67	2.15		87.78	10.05	2.16		97.09	2.91	
**Sibling’s parental status**				6.33 (Pr = 0.042)				70.71 (Pr = 0.000)			53.80 (Pr = 0.000)
Sibling is childless	77.64	18.31	4.05		77.70	15.22	7.08		91.28	8.72	
At least 1 child	80.73	16.89	2.38		87.18	11.15	1.67		97.38	2.62	
**Distance between siblings (km)**				162.99 (Pr = 0.000)				119.53 (Pr = 0.000)			71.59 (Pr = 0.000)
0–5	63.62	28.38	8.00		74.57	18.55	6.88		90.75	9.25	
6–10	77.36	17.30	5.35		81.90	13.65	4.44		95.24	4.76	
11–20	81.12	15.63	3.24		84.48	12.24	3.28		95.54	4.46	
21–50	80.58	17.27	2.16		84.59	12.19	3.23		96.77	3.23	
51–100	82.37	15.96	1.67		87.55	10.97	1.49		97.77	2.23	
101+	84.88	14.27	0.85		90.03	9.35	0.62		98.20	1.80	

*Note*. Pr = Significance value for Pearson chi-square tests.

The mean distance in our sample of sibling dyads was 211.9 km (*SE* = 552.9 km). The observed minimum distance to a sibling was 0 km, while the maximum was 13,000 km. Only in 14 dyads did the index person reported 0 km as the distance, which likely indicates co-residence. Based on Gentrans data, around 34% of older adults had at least one sibling within 10 km—a very similar estimate was found for Sweden based on register data for the whole population of older adults aged 65–84 years (Artamonova & Gillespie, 2023).

To assess differences in the frequency of help exchange and the likelihood of providing a sibling with personal care by the categories of the main explanatory variables, Table 1 presents bivariate results. Overall, there was a statistically significant association between the explanatory variables and almost all indicators of support exchange (*p* *<* .001 for all Pearson χ^2^ tests); the only exception was the non-significant relationships between index persons’ parental status combined with child’s proximity and giving care to a sibling (χ^2^ = 4.5319, *p* = .104). The bivariate results suggest that unpartnered and childless individuals receive and give intragenerational support more frequently, and the closer siblings live to each other, the greater the frequency and likelihood of support exchange.

### Regression results

**Figure 1:**
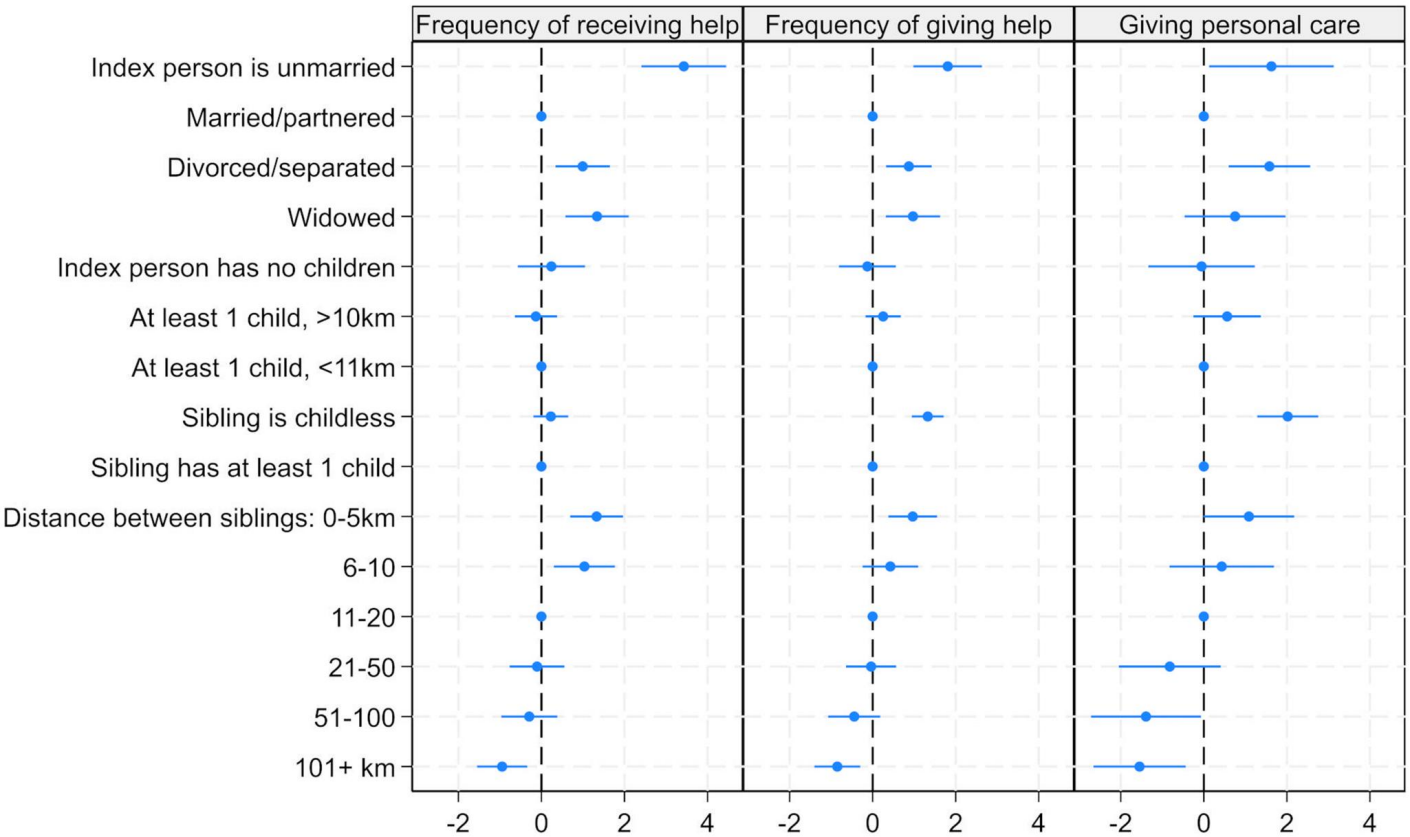
Multilevel ordinal logistic regression results for the main explanatory variables and frequency of receiving practical help from siblings and giving it to siblings as well as logistic regression results for the likelihood of giving personal care to siblings, coefficients, and 95% confidence intervals. All models control for the index person’s subjective health, language, sibling’s age, sibship size, having at least one parent alive, urbanity of index’s municipality, gender composition of a dyad, type of sibling ties, relative subjective economic situation, emotional closeness. Full models are presented in Table S2.

In line with *Hypothesis 1*, Figure 1 shows that relative to having a partner (through cohabitation, marriage, or registered partnership), being divorced, widowed, or unmarried was associated with a higher frequency of both receiving practical help from and giving it to a sibling, as well as a higher likelihood of providing personal care to a sibling (all coefficients and SEs are presented in Table S2). However, the association between being widowed and the likelihood of giving personal care to a sibling was not statistically significant.

We further hypothesized (*Hypothesis 2*) that childless individuals or those with no children nearby would give and receive practical help from siblings more frequently and would be more likely to give personal care to siblings than those who have a child nearby or at all. While the bivariate analyses suggest some support for this hypothesis, our models do not point to a statistically significant difference in the frequencies of intragenerational practical help exchange between index persons with a child nearby, those whose closest child lived more than 10 km away, and the childless (an additional check indicated that marital status explains this bivariate relationship between support and parental status). Neither did we find differences in the frequency of receiving help from siblings with or without children. However, in partial support for Hypothesis 2, we found that the frequency of giving practical help and the likelihood of providing personal care were higher if the sibling in a dyad was childless.

**Figure 2:**
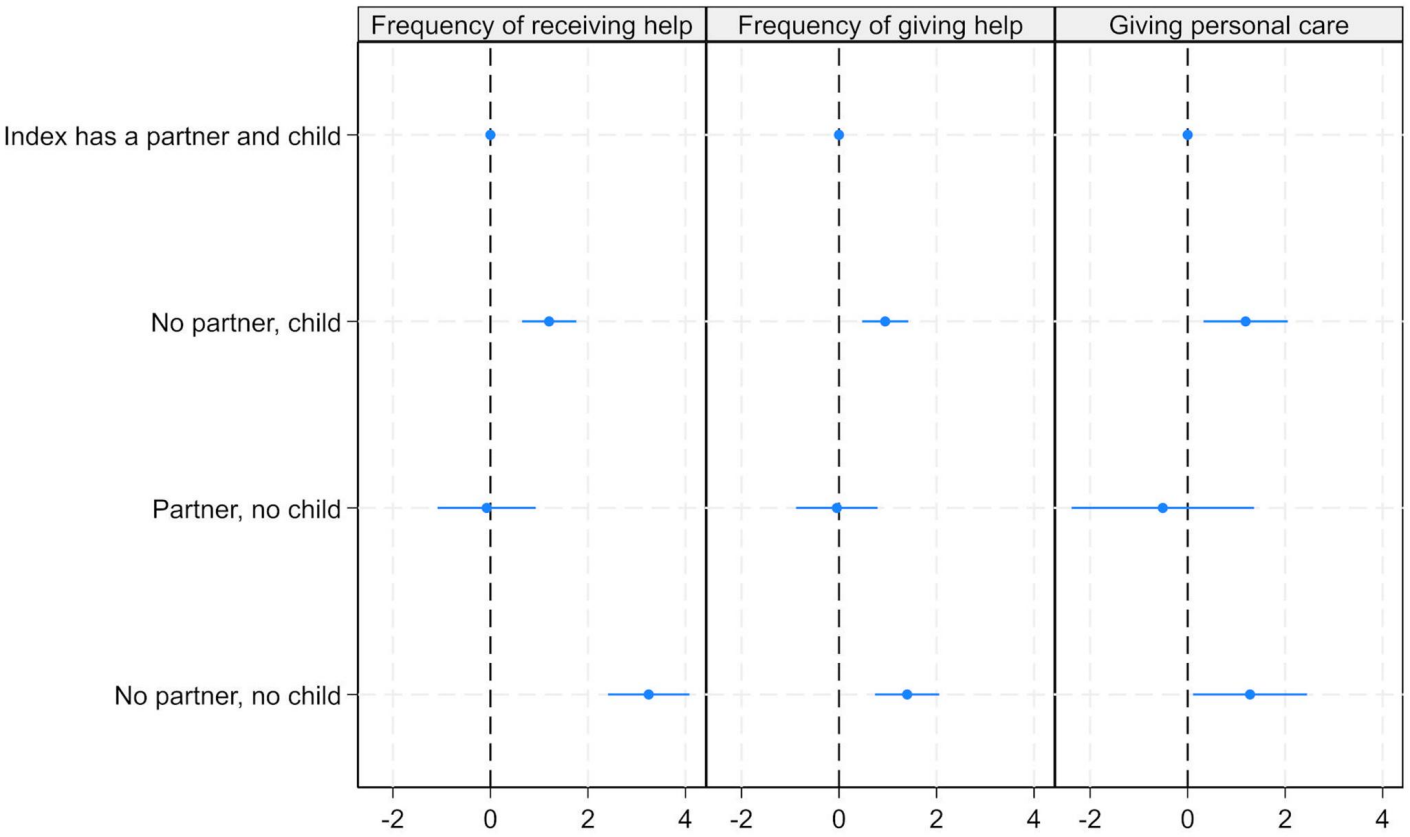
Multilevel ordinal logistic regression results for the frequency of receiving practical help from siblings and giving it to siblings as well as logistic regression results for the likelihood of giving personal care to siblings, coefficients, and 95% confidence intervals for core-kinlessness. All models control for the index person’s subjective health, language, sibling’s age, sibship size, having at least one parent alive, urbanity of index’s municipality, gender composition of a dyad, type of sibling ties, relative subjective economic situation, and emotional closeness. All estimates are presented in Table S3. Average predicted probabilities from this model are also presented in Figure S1.

As for the interplay between lacking a partner and children, in line with *Hypothesis 3*, Figure 2 illustrates that not having a partner and children (relative to having both types of core family members) was associated with a higher frequencies of receiving practical help from siblings (B = 3.247, *p* *<* .001) and giving practical help to siblings (B = 1.398, *p* < .001), as well as the higher likelihood of providing siblings with personal care (B = 1.279, *p* < .01). Similar results were found for lacking only a partner, but not for lacking a child as shown in Figure 1.

In line with *Hypothesis 4*, Figure 1 shows that relative to those who lived at the distance of 11–20 km from a sibling, older adults receive help more frequently if they lived within 10 km of a sibling and less frequently it if the intragenerational distance exceeded 100 km. Relative to the same reference category (11–20 km), living within 0–5 km of a sibling was associated with a higher frequency of giving practical help to a sibling and living more than 100 km away was associated with a lower frequency. A similar trend was found for the likelihood of providing a sibling with personal care, although this likelihood decreased at distances beyond 51 km. Based on these findings, the 10 km threshold that has previously been used in research based on register data to approximate help and care exchange between siblings in Sweden (e.g., Artamonova & Gillespie, 2023) seems adequate for intragenerational exchanges and can be used in Finland and, perhaps, in other Nordic countries. We, therefore, adhere to this operationalization of close distance to explore the interaction effect between lacking core family members and intragenerational proximity in predicting help exchange and personal care provision.

**Figure 3:**
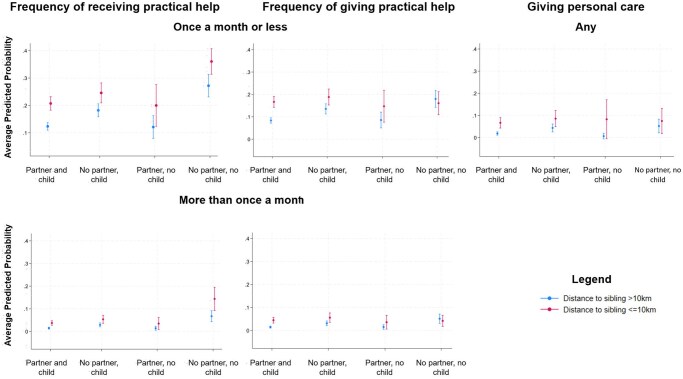
Multilevel ordinal logistic regression results for the variations in the effects of the geographic proximity on frequency of receiving practical help from siblings, giving it to siblings, and giving any personal care to siblings by the availability of index person’s core family members, average predicted probabilities and 95% confidence intervals. All models control for the index person’s subjective health, language, sibling’s age, sibship size, having at least one parent alive, urbanity of index’s municipality, gender composition of a dyad, type of sibling ties, relative subjective economic situation, and emotional closeness.

Finally, we expected the association between support exchange and distance to be weaker for core-kinless index persons than for those with core kin. Figure 3 shows that distance makes a greater difference in intragenerational support exchange for those who have both a partner and children than for those who lack a partner, a child, or both. More specifically, our model suggested that among siblings living more than 10 km apart, core-kinless index persons were more likely to receive help both once a month and more frequently than those with at least one child but without a partner, those with a partner but without children, and those with both a partner and a child (Figure 3, left panel). However, the proximity still mattered: The APPs of receiving help by core-kinless index persons within both non-zero frequency categories were significantly higher if a sibling lived within 10 km (0.360 for monthly or less and 0.144 for more than monthly) than outside 10 km radius (0.273 and 0.067 respectively). We further found that among those who lived more than 10 km apart, core-kinless older adults provided practical help to their siblings more frequently than those with a partner or both a partner and at least one child, while no significant differences were observed among those who lived close (center panel). In line with our expectation, the frequencies of providing practical help to a sibling by core-kinless index persons were equally high among those who lived close to (APPs equaled 0.161 for monthly or less and 0.042 for more than monthly) and far from a sibling (0.180 for monthly or less and 0.051 for more than monthly). Also in line with our expectation, the APPs of giving personal care did not differ between core-kinless older adults living far from (0.052) and close to (0.075) a sibling and were higher than distant siblings with a partner (right panel). In additional analyses, we explored the interaction effect between siblings’ parenthood status and proximity (Figure S2).

Our results for control variables are largely consistent with previous research and described in Supplementary Material.

## Discussion

In the light of ongoing debates on family complexity and the importance of family members beyond a partner and children for support exchange, we aimed to explore the role of siblings, especially for older adults who are core-kinless. We show that siblings generally support each other in later life even in the less familialistic context of Finland. In line with theories of social support and family solidarity, including the hierarchical-compensatory model (Cantor, 1991), the functional specificity model (Connidis, 1994), and the convoy model (Kahn & Antonucci, 1980), our findings demonstrate that the absence of core family members shapes the dynamics of sibling support in later life.

Partnerless older adults, especially those who never married, were significantly more likely to receive practical help from siblings than their partnered counterparts. This finding is in line with the functional specificity of relationships model suggesting that the never-married could establish supportive ties with siblings in earlier life course stages, while those who were married and remained married or lost their partners might have a different system of support (Connidis, 1994). While we expected that childlessness would similarly increase the likelihood of receiving support from siblings, the lack of significant differences based on the proximity or presence of children suggests that sibling support is primarily responsive to the absence of a partner rather than of children. However, we found that older adults without both a partner and children were the most likely to receive help from siblings. These findings align with prior research suggesting that siblings can serve as substitute caregivers when more normative sources are unavailable (Fihel et al., 2021) and underscore the adaptive and flexible nature of family roles across the life course, including later life.

We also examined the support provided by core-kinless older adults to their siblings. We found that core-kinless individuals not only received more help but also gave more practical assistance and personal care to siblings than those with partners or children. This finding is in line with the convoy model suggesting that core-kinless older adults may redirect their social investments toward existing horizontal ties. Importantly, core-kinless individuals provided high levels of support to their siblings regardless of geographic distance. This suggests a compensatory motivation that may outweigh logistical constraints.

Surprisingly and contrary to previous research showing that people tend to overreport help they give and underreport help they receive from parents and children (Mandemakers & Dykstra, 2008) or spouses (Rodrigues et al., 2024), Gentrans respondents reported more help received from siblings than help provided to siblings. This result could be explained by our asymmetrical sample of respondents and siblings: While siblings could be “old-old” or rather young, respondents’ age was fixed.

We further explored the role of geographic proximity between siblings in support exchange. Our results are generally in line with previous findings on the “distance decay” effect (e.g., Knijn & Liefbroer, 2006). Both giving and receiving support were significantly more frequent among siblings who lived within 10 km of each other. The consistency of this threshold across outcomes validates its use as a proxy for support availability in register-based studies, particularly in the Nordic context. However, it should be noted that core-kinless older adults still received more help than their peers with core family members across both distance categories. While proximity enhances the likelihood and frequency of support, it does not entirely determine it.

Control variables add nuances to our results about sibling support exchange. Emotional closeness, gender composition, age similarity, and relative income played roles in shaping support exchanges. Interestingly, personal care was more likely to be provided to older, low-income siblings, indicating that need-based factors shape support flows. This responsiveness to needs further reinforces the flexibility and functional specificity of sibling relationships in later life. Surprisingly, none of the health measures available in Gentrans was significantly associated with sibling support exchange, which might be explained by the relatively early stage of index persons’ aging.

Our research contributes to the literature on informal support in later life in several ways. First, we explored the role of older adults’ siblings—family members who are often overlooked in research about later-life caregiving. Our findings emphasize the supportive nature of sibling bonds. Second, while previous research on support exchange between siblings in later life was largely conducted either quite long ago (e.g., Avioli, 1989), based on register data where support was approximated via geographic proximity (e.g., Artamonova & Gillespie, 2023), or based on qualitative data (e.g., Sýkorová, 2024), our study utilizes relatively recent data from Finland that include information on multiple aspects of older adults’ life rarely captured in a single survey: information on family members beyond a partner and children, support exchange between siblings, intragenerational geographic distance, emotional closeness, and health. As the population ages and core-kinlessness rises, siblings may become more central figures in informal caregiving networks than studies from the previous century suggested. Third, our results challenge the assumption that older adults without partners and children are passive recipients of care. We demonstrated that these older adults are also active providers of help and care. This result invites reframing of how core-kinless older adults are understood in gerontological literature. Future research and policy should recognize core-kinless older adults as an underappreciated resource of help and care within family networks. Finally, we empirically confirmed that researchers seeking to approximate help and care exchange between siblings can continue utilizing the 10 km distance threshold.

Despite offering novel insights into social support literature, this study has several limitations. First, we did not have information on receiving personal care from siblings, or siblings’ partnership status, health, and urbanity of their place of residence. These variables would enrich our understanding of intragenerational exchanges in later life. Second, index persons were not old enough to require extensive personal care and, therefore, we could neither fully estimate their siblings’ caregiving potential nor formally compare the patterns of exchanging help and care by intragenerational distance and availability of core kin. Future research could address these limitations and explore the quality and emotional dimensions of sibling exchanges, as well as the longitudinal stability of caregiving roles, and differences in the exchange of practical help and personal care between siblings among the oldest-old. Qualitative insights could enrich our understanding of how siblings negotiate caregiving expectations and obligations over time, particularly in the context of declining health and increased dependency.

Our findings have some important policy implications. First, policymakers should broaden caregiving definitions in policy and planning to include nontraditional care networks such as siblings. This could include, for example, extending caregiver benefits (e.g., tax credits, respite care, paid leave) to siblings, especially those with low income, as well as acknowledging the diversity of family caregiving arrangements in later life and including sibling caregivers in the design and outreach of support programs. Second, although geographic proximity strongly relates to caregiving, some core-kinless older adults appear to provide or receive care from siblings regardless of distance, suggesting high motivation but potentially high burden. Policymakers could develop services to support long-distance caregiving, such as, for instance, subsidized travel or caregiving leaves for siblings. Alternatively, relocation services could assist older adults, especially the core-kinless, in migration toward siblings, reduce the costs of these migrations, and, ideally, ensure the desired types of housing with amenities nearby if siblings live far apart but are willing to move closer to become caregivers (Artamonova & Gillespie, 2022). Third, policies and services aimed at supporting core-kinless older adults should not only address this group’s increased care needs but also recognize their caregiving potential. On a more general note, lifelong sibling bonds should be encouraged via public health initiatives that promote strong family connections beyond the nuclear family.

## Supplementary Material

gbag108_Supplementary_Data

## Data Availability

Information about the Gentrans project can be found here: https://www.vaestoliitto.fi/en/research/ageing-and-generations/generational-transmissions-in-finland/, the survey dataset is available upon request from Prof. Dr. Mirkka Danielsbacka. Licenses to the use of Finnish data linked to the Gentrans 2018 are granted by Statistics Finland (https://stat.fi/). The analytic code is not available publicly as the first author has not completed planned analyses for all publications on her project but can be shared upon request. The study is not pre-registered.

## References

[gbag108-B1] Artamonova A. , Gillespie B. J. (2022). Older adults’ internal migration toward faraway siblings. The Journals of Gerontology, Series B: Psychological Sciences and Social Sciences, 77, 1336–1349. 10.1093/geronb/gbac011 35137067 PMC9255943

[gbag108-B2] Artamonova A. , Gillespie B. J. (2023). Geographic proximity to siblings in older adulthood. Demographic Research, 49, 143–156. 10.4054/DemRes.2023.49.7

[gbag108-B3] Avioli P. S. (1989). The social support functions of siblings in later life: A theoretical model. American Behavioral Scientist, 33, 45–57. 10.1177/0002764289033001005

[gbag108-B4] Brandt M. , Haberkern K., Szydlik M. (2009). Intergenerational help and care in Europe. European Sociological Review, 25, 585–601. 10.1093/esr/jcn076

[gbag108-B5] Buchanan A. , Rotkirch A. (Eds.). (2021). Brothers and sisters: Sibling relationships across the life course. Palgrave Macmillan. 10.1007/978-3-030-55985-4

[gbag108-B6] Cantor M. H. (1991). Family and community: Changing roles in an aging society. The Gerontologist, 31, 337–346. 10.1093/geront/31.3.337 1879709

[gbag108-B7] Cattaneo A. , Vitali A., Regazzoni D., Rizzi C. (2025). The burden of informal family caregiving in Europe, 2000-2050: A microsimulation modelling study. The Lancet Regional Health. Europe, 53, 101295. 10.1016/j.lanepe.2025.101295 40255934 PMC12008708

[gbag108-B8] Connidis I. A. (1994). Sibling support in older age. Journal of Gerontology, 49, S309–S317. 10.1093/geronj/49.6.S309 7963288

[gbag108-B10] Connidis I. A. , Barnett A. E. (2018). Family ties and aging. Sage. 10.4135/9781544342306

[gbag108-B12] Dykstra P. , Knipscheer K. (1995). The availability and intergenerational structure of family relationships. In Knipscheer C. P. M., de Jong Gierveld J., van Tilburg T. G., Dykstra P. A. (Eds.), Living arrangements and social networks of older adults. VU University Press.

[gbag108-B13] Einiö E. (2010). Determinants of institutional care at older ages in Finland. Finnish Yearbook of Population Research, 45, 1–139. 10.23979/fypr.45288

[gbag108-B14] Einiö E. , Nisén J., Martikainen P. (2016). Number of children and later-life mortality among Finns born 1938-50. Population Studies, 70, 217–238. 10.1080/00324728.2016.1195506 27362776

[gbag108-B15] Eriksen S. , Gerstel N. (2002). A labor of love or labor itself: Care work among adult brothers and sisters. Journal of Family Issues, 23, 836–856. 10.1177/019251302236597

[gbag108-B17] Gilligan M. , Stocker C. M., Jewsbury Conger K. (2020). Sibling relationships in adulthood: Research findings and new frontiers. Journal of Family Theory & Review, 12, 305–320. 10.1111/jftr.12385

[gbag108-B18] Gold D. T. (1987). Siblings in old age: Something special. Canadian Journal on Aging / La Revue Canadienne du Vieillissement, 6, 199–216. 10.1017/S0714980800008424

[gbag108-B4687820] Hicks S. A., , Horowitz A., , Jimenez D., , Falzarano F., , Minahan J., & , Cimarolli V. R. (2018). Unique challenges reported by long-distance caregivers. Innovation in Aging, 2, 201–201. 10.1093/geroni/igy023.739

[gbag108-B20] Hyyppa M. T. (2010). Successful aging of Swedish-speaking Minority Finns. The role of social capital. In Isomaki V-P., Health, Wellness and Social Policy. Essays in honour of Guy Bäckman (pp. 80–94). Europ.

[gbag108-B21] Kahn R. L. , Antonucci T. C. (1980). A life course approach. In Baltes P. B., Brim O. G. (Eds.), Convoys over the life course: Life span development and behavior (pp. 253–286). Academic Press.

[gbag108-B22] Kalmijn M. , Leopold T. (2019). Changing sibling relationships after parents’ death: The role of solidarity and kinkeeping. Journal of Marriage and Family, 81, 99–114. 10.1111/jomf.12509

[gbag108-B23] Kalmijn M. , Saraceno C. (2008). A comparative perspective on intergenerational support: Responsiveness to parental needs in individualistic and familialistic countries. European Societies, 10, 479–508. 10.1080/14616690701744364

[gbag108-B24] Kangas O. , Kvist J. (2018). Nordic welfare states. In Greve B. (Eds.), Routledge handbook of the welfare state (pp. 124–136). Routledge.

[gbag108-B26] Knijn T. C. , Liefbroer A. C. (2006). More kin than kind: Instrumental support in families. In P. A Dykstra, M Kalmijn, T. C. M Knijn, A. E Komter, A. C Liefbroer, C. H Mulder. (Eds.), Family solidarity in the Netherlands (pp. 89–105). Dutch University Press.

[gbag108-B27] Korhonen K. , Einiö E., Martikainen K. (2026). The association of number and geographic proximity of children with care home use before all-cause and dementia deaths: A register-based study of Finnish older adults. The Journals of Gerontology, Series B: Psychological Sciences and Social Sciences, 81, gbaf250. 10.1093/geronb/gbaf250 41369121 PMC12795604

[gbag108-B28] Leitner S. (2003). Varieties of familialism: The caring function of the family in comparative perspective. European Societies, 5, 353–375. 10.1080/1461669032000127642

[gbag108-B29] Lopata H. (2017). Widowhood in an American city. Routledge.

[gbag108-B30] Malmberg G. , Pettersson A. (2007). Distance to elderly parents: Analyses of Swedish register data. Demographic Research, 17, 679–704. 10.4054/DemRes.2007.17.23

[gbag108-B31] Mandemakers J. J. , Dykstra P. A. (2008). Discrepancies in parent’s and adult child’s reports of support and contact. Journal of Marriage and Family, 70, 495–506. 10.1111/j.1741-3737.2008.00496.x

[gbag108-B32] Margolis R. , Verdery A. M. (2017). Older adults without close kin in the United States. The Journals of Gerontology, Series B: Psychological Sciences and Social Sciences, 72, 688–693. 10.1093/geronb/gbx068 28575387 PMC5927096

[gbag108-B33] Marois G. , Rotkirch A., Lutz W. (2022). Future population ageing and productivity in Finland under different education and fertility scenarios. Finnish Yearbook of Population Research, 55, 137–160. 10.23979/fypr.119666

[gbag108-B34] Mulder C. H. , Van der Meer M. J. (2009). Geographical distances and support from family members. Population, Space and Place, 15, 381–399. 10.1002/psp.557

[gbag108-B35] Nicolaisen M. , Thorsen K. (2014). Who are lonely? Loneliness in different age groups (18-81 years old), using two measures of loneliness. International Journal of Aging & Human Development, 78, 229–257. 10.2190/AG.78.3.b 25265679

[gbag108-B36] Ong A. D. , Uchino B. N., Wethington E. (2016). Loneliness and health in older adults: A mini-review and synthesis. Gerontology, 62, 443–449. 10.1159/000441651 26539997 PMC6162046

[gbag108-B37] Pani-Harreman K. E. , Bours G. J., Zander I., Kempen G. I., van Duren J. M. (2021). Definitions, key themes and aspects of “ageing in place”: A scoping review. Ageing and Society, 41, 2026–2059. 10.1017/S0144686X20000094

[gbag108-B38] Patterson S. E. , Margolis R., Verdery A. M. (2020). Family embeddedness and older adult mortality in the United States. Population Studies, 74, 415–435. 10.1080/00324728.2020.1817529 33016247 PMC7642151

[gbag108-B39] Pavolini E. , Ranci C. (2008). Restructuring the welfare state: Reforms in long-term care in Western European countries. Journal of European Social Policy, 18, 246–259. 10.1177/0958928708091058

[gbag108-B40] Plick N. P. , Ankuda C. K., Mair C. A., Husain M., Ornstein K. A. (2021). A national profile of kinlessness at the end of life among older adults: Findings from the health and retirement study. Journal of the American Geriatrics Society, 69, 2143–2151. 10.1111/jgs.17171 33880751 PMC8373783

[gbag108-B41] Rodrigues R. , Simmons C., Zólyomi E., Vafaei A., Rehnberg J., Kadi S., Socci M., Fors S., Phillips S. P. (2024). Depends on whom you ask: Discordance in reporting spousal care between older women and men across European welfare states. Archives of Gerontology and Geriatrics, 125, 105518. 10.1016/j.archger.2024.105518 38876081

[gbag108-B42] Rossi A. S. , Rossi P. H. (1990). Of human bonding: Parent-child relations across the life course. Routledge.

[gbag108-B43] Rostgaard T. , Jacobsen F., Kröger T., Peterson E. (2022). Revisiting the Nordic long-term care model for older people—Still equal? European Journal of Ageing, 19, 201–210. 10.1007/s10433-022-00703-4 35528216 PMC9062627

[gbag108-B44] Rotkirch A. (2021). Executive summary of the Finnish population policy report “Recovery of the birth rate and longer life expectancy-population policy guidelines for the 2020s.” Prime Minister’s Office, Finland 2021:2.

[gbag108-B45] Rotkirch A. , Berg V., Finnäs F. (2018). Svenskspråkigas fruktsamhet i Finland. Vilka är skillnaderna mellan språkgrupperna.

[gbag108-B46] Saarela J. , Finnäs F. (2018). Ethno-linguistic exogamy and divorce: Does marital duration matter? Sociological Focus, 51, 279–303. 10.1080/00380237.2018.1431506

[gbag108-B47] Shanas E. (1979). The family as a social support system in old age. The Gerontologist, 19, 169–174. 10.1093/geront/19.2.169 263596

[gbag108-B48] Simons R. L. (1983-1984). Specificity and substitution in the social networks of the elderly. International Journal of Aging & Human Development, 18, 121–139. 10.2190/AUY4-CMPK-JFCB-E04V 6668076

[gbag108-B49] Stocker C. M. , Gilligan M., Klopack E. T., Conger K. J., Lanthier R. P., Neppl T. K., O’Neal C. W., Wickrama K. A. S. (2020). Sibling relationships in older adulthood: Links with loneliness and well-being. Journal of Family Psychology, 34, 175–185. 10.1037/fam0000586 31414866 PMC7012710

[gbag108-B50] Sýkorová D. (2024). Siblinghood amongst older adults: What being a sibling or having siblings means. Journal of Family Issues, 45, 419–446. 10.1177/0192513X221150974

[gbag108-B51] Syse A. , Artamonova A., Thomas M., Veenstra M. (2022). Do characteristics of family members influence older persons’ transition to long-term healthcare services? BMC Health Services Research, 22, 362. 10.1186/s12913-022-07745-5 35303891 PMC8933970

[gbag108-B52] Tanskanen A. O. , Rotkirch A. (2019). Sibling similarity and relationship quality in Finland. Acta Sociologica, 62, 440–456. 10.1177/0001699318777042

[gbag108-B53] Tanskanen A. O. , Danielsbacka M., Rotkirch A. (2021). Kin detection cues and sibling relationship quality in adulthood: The role of childhood co-residence duration and maternal perinatal association. Evolution and Human Behavior, 42, 481–490. 10.1016/j.evolhumbehav.2021.05.004

[gbag108-B54] Terhell E. L. , Broese van Groenou M. I., van Tilburg T. (2004). Network dynamics in the long-term period after divorce. Journal of Social and Personal Relationships, 21, 719–738. 10.1177/0265407504047833

[gbag108-B55] Thomas M. J. , Dommermuth L. (2020). Internal migration and the role of intergenerational family ties and life events. Journal of Marriage and Family, 82, 1461–1478. 10.1111/jomf.12678

[gbag108-B6323154] Verdery A. M., , Margolis R., , Zhou Z., , Chai X., & , Rittirong J. (2019). Kinlessness Around the World. The Journals of Gerontology, Series B: Psychological Sciences and Social Sciences, 74, 1394–1405. 10.1093/geronb/gby138 30423167 PMC6777763

[gbag108-B56] White L. (2001). Sibling relationships over the life course: A panel analysis. Journal of Marriage and Family, 63, 555–568. 10.1111/j.1741-3737.2001.00555.x

