# Supplementary material for: Support exchange between siblings in later life: the role of “core-kinlessness” and geographic proximity
Source: J Gerontol B Psychol Sci Soc Sci. 2026 Jun 20;81(9):gbag108. doi: 10.1093/geronb/gbag108 (PMC13439929; doi:10.1093/geronb/gbag108)
Supplement: Supplementary file 1 [file gbag108_supplementary_data.docx]

***The Journals of Gerontology, Series B: Psychological Sciences and Social Sciences* Supplementary Material: Artamonova et al. Support exchange between siblings in later life: The role of** **‘core-kinlessness’ and geographic proximity**

**Table S1**. Characteristics of the sample

|  | Mean (SE) **or %** |
| --- | --- |
| **Index’s marital status** |  |
| Unmarried/unpartnered | 7.4 |
| Married/partnered | 67.0 |
| Divorced/separated | 16.4 |
| Widowed | 9.2 |
| **Index’s parental status and child’s proximity** |  |
| Index person has no children | 13.2 |
| At least 1 child, >10km | 40.9 |
| At least 1 child, <11km | 45.9 |
| **Sibling’s parental status** |  |
| Sibling has no children | 14.0 |
| At least 1 child | 86.0 |
| **Distance between siblings** |  |
| 0–5 km | 12.9 |
| 6–10 | 7.8 |
| 11–20 | 8.4 |
| 21–50 | 13.7 |
| 51–100 | 13.3 |
| 101+ km | 43.9 |
| **Index’s subjective health** |  |
| Good | 51.7 |
| Fair | 43.6 |
| Poor | 4.7 |
| **Index's language (Ref: Finnish)** |  |
| Finnish | 95.8 |
| Swedish | 4.2 |
| **Index’s urbanity of municipality** |  |
| Urban | 67.88 |
| Semi-urban municipality | 17.65 |
| Rural municipality | 14.47 |
| **Characteristics of Index person-sibling dyad** |  |
| **Gender composition** |  |
| Sister-sister | 32.7 |
| Sister-brother | 28.0 |
| Brother-sister | 20.3 |
| Brother-brother | 19.0 |
| **Sibling ties** |  |
| Full siblings | 94.6 |
| Same mother only | 3.7 |
| Same father only | 1.7 |
| **Emotional closeness between siblings** | 3.7 (0.94) |
| **Sibling’s age** |  |
| Sibling younger than 60 years old | 8.2 |
| 61–65 | 17.1 |
| 66–75 | 52.4 |
| 76–80 | 14.5 |
| ≥81 years old | 7.7 |
| **Subjective relative economic situation** |  |
| Both with middle or higher | 47.7 |
| Index with middle+, sib with low | 8.9 |
| Index with low, sib with middle+ | 30.3 |
| Both with low | 13.1 |
| **Parental vital status** |  |
| At least one parent alive | 11.0 |
| No parents alive | 89.0 |
| **Initial sibship size** | 3.8 (1.31) |
| **N of dyads** | 4,050 |
| **N of index persons** | 1,699 |

*Note*: the estimates are given for the sample used for modelling the outcome ´receiving practical help´. The distributions used for modelling other outcomes are not different (variation is within 0.1).

***Results for control variables***

Our results for control variables are largely consistent with previous research (Table S2). Emotional closeness between siblings was a strong predictor of all outcomes. Living in semi-urban or rural municipality (relative to an urban area) was associated with a higher frequency of help exchange and a higher likelihood of giving personal care. Support of all explored types was more likely to be given (at all or more frequently) to older siblings and received from the younger ones. Having at least one parent alive was marginally associated with a higher frequency of receiving help and a higher likelihood of providing care. Index person’s subjective health, language group, and type of sibling tie (after controlling for emotional closeness, there is no difference between full and half siblings) were not significantly associated with either the frequency of practical help exchange or the likelihood of providing personal care.

Other factors were outcome-specific. Relative to dyads of two sisters, sisters were less likely to receive practical help and personal care from brothers. A similar result appeared for brothers receiving personal care from sisters. Brothers provided practical help more frequently to each other and to sisters relative to dyads consisting of two sisters. Index persons received practical help from a sibling in a dyad more frequently if both siblings had middle or high subjective income relative to all other income combinations. Compared to dyads of two financially well-off siblings, index persons provided siblings with help more frequently and were more likely to provide them with care if siblings had low income and were less likely to do so if index persons themselves had lower subjective income than their sibling. Ever having had many siblings was associated with a lower frequency of receiving and giving practical help to a sibling in a dyad but was not related to the likelihood of providing a sibling with personal care.

***Sensitivity analyses***

We also explored the robustness of our models using a distance categorization similar to the one employed in the Survey of Health, Ageing and Retirement in Europe. Relative to an intragenerational distance of 6-25 km, a shorter distance was associated with a higher frequency of help exchange and a higher likelihood of care provision, while a longer distance was associated with lower frequencies and a lower likelihood of the respective support outcomes (Figure S3). Another sensitivity check concerned the use of alternative indicators of the index person’s health. None of the support exchange outcomes were associated with i) having any long-term health issue, disease, or permanent disability or ii) receiving a disability pension. We also employed an extended sample that includes individuals with missing values in control variables (Table S2); the results remained largely consistent with the main models.

**Table S2.** Models with and without control variables, as well as models with missing values in control variables

|  | **Frequency of receiving practical help** | | | **Frequency of giving practical help** | | | | | **Giving personal care** | | | | |
| --- | --- | --- | --- | --- | --- | --- | --- | --- | --- | --- | --- | --- | --- |
|  | **Without controls** | **With controls** | **With missings in controls** | **Without controls** | **With controls** | **With missings in controls** | | **Without controls** | | **With controls** | | **With missings in controls** | |
| **Index’s marital status (Ref: married/partnered)** | | | |  |  |  | |  | |  | |  | |
| Unmarried/unpartnered | 2.492*** | 3.429*** | 3.005*** | 1.373*** | 1.808*** | 1.802*** | | 1.285* | | 1.628* | | 1.707* | |
|  | (0.428) | (0.521) | (0.489) | (0.378) | (0.422) | (0.410) | | (0.607) | | (0.766) | | (0.707) | |
| Divorced/separated | 0.393 | 0.994** | 0.882** | 0.270 | 0.872** | 0.861** | | 1.125** | | 1.579** | | 1.493** | |
|  | (0.265) | (0.334) | (0.316) | (0.248) | (0.280) | (0.269) | | (0.373) | | (0.502) | | (0.458) | |
| Widowed | 1.034** | 1.339** | 1.433*** | 0.625* | 0.972** | 0.750* | | 0.748 | | 0.753 | | 0.589 | |
|  | (0.322) | (0.391) | (0.361) | (0.301) | (0.334) | (0.320) | | (0.476) | | (0.621) | | (0.556) | |
| **Index’s parental status and child’s proximity (Ref: child within 10 km)** | | | | | | |  | |  | |  | |  |
| Index person has no children | 0.506 | 0.240 | 0.592 | 0.123 | -0.127 | -0.149 | | -0.068 | | -0.054 | | -0.0913 | |
|  | (0.339) | (0.414) | (0.387) | (0.324) | (0.350) | (0.338) | | (0.526) | | (0.656) | | (0.596) | |
| At least 1 child, >10km | -0.013 | -0.133 | -0.0210 | 0.341† | 0.253 | 0.203 | | 0.526† | | 0.562 | | 0.439 | |
|  | (0.212) | (0.259) | (0.243) | (0.198) | (0.218) | (0.208) | | (0.318) | | (0.416) | | (0.375) | |
| **Sibling’s parental status (Ref: sibling has a child)** | | | | | | |  | |  | |  | |  |
| Childless sibling | 0.184 | 0.227 | 0.158 | 1.084*** | 1.327*** | 1.320*** | | 1.487*** | | 2.019*** | | 1.903*** | |
|  | (0.179) | (0.215) | (0.201) | (0.174) | (0.196) | (0.185) | | (0.277) | | (0.376) | | (0.336) | |
| **Distance between siblings (Ref: 11-20 km)** | | | |  |  |  | |  | |  | |  | |
| 0–5 km | 1.331*** | 1.329*** | 1.126*** | 1.116*** | 0.966** | 1.017*** | | 0.914* | | 1.086† | | 1.276* | |
|  | (0.277) | (0.325) | (0.295) | (0.277) | (0.300) | (0.281) | | (0.440) | | (0.556) | | (0.518) | |
| 6–10 | 0.796* | 1.035** | 0.769* | 0.441 | 0.426 | 0.648* | | 0.131 | | 0.432 | | 0.687 | |
|  | (0.320) | (0.376) | (0.345) | (0.316) | (0.342) | (0.323) | | (0.515) | | (0.642) | | (0.594) | |
| 21–0 | 0.160 | -0.105 | -0.255 | 0.051 | -0.0370 | 0.0355 | | -0.644 | | -0.820 | | -0.574 | |
|  | (0.288) | (0.337) | (0.309) | (0.286) | (0.308) | (0.292) | | (0.500) | | (0.626) | | (0.577) | |
| 51–100 | -0.286 | -0.293 | -0.423 | -0.313 | -0.443 | -0.382 | | -1.282* | | -1.393* | | -1.065† | |
|  | (0.292) | (0.345) | (0.317) | (0.295) | (0.321) | (0.301) | | (0.552) | | (0.676) | | (0.605) | |
| 101+ km | -0.654** | -0.946** | -1.114*** | -0.671** | -0.851** | -0.872** | | -1.419** | | -1.547** | | -1.382** | |
|  | (0.262) | (0.309) | (0.283) | (0.259) | (0.282) | (0.267) | | (0.453) | | (0.568) | | (0.521) | |
| **Index’s subjective health (Ref: good)** | | | |  |  |  | |  | |  | |  | |
| Fair |  | -0.027 | -0.048 |  | -0.095 | -0.180 | |  | | 0.312 | | 0.363 | |
|  |  | (0.246) | (0.232) |  | (0.207) | (0.198) | |  | | (0.392) | | (0.360) | |
| Poor |  | -0.528 | -0.475 |  | -1.021† | -1.176* | |  | | 0.325 | | 0.533 | |
|  |  | (0.627) | (0.575) |  | (0.545) | (0.522) | |  | | (0.849) | | (0.742) | |
| Unknown |  |  | 0.232 |  |  | -3.106† | |  | |  | | -0.705 | |
|  |  |  | (1.241) |  |  | (1.881) | |  | |  | | (1.819) | |
| **Index’s language (Ref: Finnish)** | |  |  |  |  |  | |  | |  | |  | |
| Swedish |  | -0.555 | -0.426 |  | 0.0830 | 0.191 | |  | | -1.465 | | -1.633 | |
|  |  | (0.544) | (0.518) |  | (0.453) | (0.437) | |  | | (1.186) | | (1.152) | |
| **Index’s urbanity of municipality (Ref: urban)** | | | |  |  |  | |  | |  | |  | |
| Semi-urban municipality |  | 0.736* | 0.691* |  | 0.902** | 0.839** | |  | | 1.064* | | 0.986* | |
|  |  | (0.317) | (0.296) |  | (0.261) | (0.250) | |  | | (0.472) | | (0.432) | |
| Rural municipality |  | 0.902** | 0.813* |  | 0.605* | 0.512† | |  | | 0.690 | | 0.487 | |
|  |  | (0.348) | (0.321) |  | (0.293) | (0.279) | |  | | (0.555) | | (0.505) | |
| **Gender composition (Ref: sister-sister)** | | |  |  |  |  | |  | |  | |  | |
| Sister-brother |  | 0.266 | 0.363* |  | -0.685*** | -0.542** | |  | | -0.735* | | -0.603† | |
|  |  | (0.193) | (0.185) |  | (0.206) | (0.195) | |  | | (0.373) | | (0.339) | |
| Brother-sister |  | 0.0821 | 0.104 |  | 0.899*** | 0.909*** | |  | | -1.178* | | -0.980* | |
|  |  | (0.298) | (0.278) |  | (0.247) | (0.236) | |  | | (0.545) | | (0.481) | |
| Brother-brother |  | 1.009** | 0.991*** |  | 1.217** | 1.176*** | |  | | -0.373 | | -0.404 | |
|  |  | (0.291) | (0.273) |  | (0.254) | (0.242) | |  | | (0.495) | | (0.453) | |
| Unknown |  |  | 1.970† |  |  | 0.873 | |  | |  | | -0.0697 | |
|  |  |  | (1.143) |  |  | (1.184) | |  | |  | | (1.901) | |
| **Sibling ties (Ref: full siblings)** | | | |  |  |  | |  | |  | |  | |
| Same mother only |  | 0.041 | -0.093 |  | 0.234 | 0.058 | |  | | 0.400 | | 0.095 | |
|  |  | (0.564) | (0.515) |  | (0.448) | (0.437) | |  | | (0.798) | | (0.704) | |
| Same father only |  | -1.360 | -0.750 |  | -1.874† | -1.173 | |  | | -1.304 | | -1.716 | |
|  |  | (1.033) | (0.806) |  | (1.057) | (0.864) | |  | | (1.755) | | (1.660) | |
| **Emotional closeness between siblings** |  | 2.312*** | 2.331*** |  | 1.521*** | 1.485*** | |  | | 1.160*** | | 1.105*** | |
|  |  | (0.164) | (0.155) |  | (0.132) | (0.123) | |  | | (0.250) | | (0.216) | |
| **Sibling’s age (Ref: 66–75 years old or close to Index’s age)** | | | |  |  |  | |  | |  | |  | |
| Sibling <60 years old |  | 0.551* | 0.616* |  | -0.420 | -0.280 | |  | | -2.743** | | -2.354** | |
|  |  | (0.273) | (0.260) |  | (0.290) | (0.279) | |  | | (1.001) | | (0.855) | |
| 61–65 |  | 0.243 | 0.228 |  | -0.113 | -0.049 | |  | | -0.552 | | -0.384 | |
|  |  | (0.193) | (0.185) |  | (0.197) | (0.191) | |  | | (0.439) | | (0.403) | |
| 76–80 |  | -0.795** | -0.886*** |  | 0.103 | 0.144 | |  | | 1.147** | | 1.123** | |
|  |  | (0.259) | (0.246) |  | (0.225) | (0.217) | |  | | (0.430) | | (0.397) | |
| ≥81 years old |  | -1.974*** | -1.684*** |  | 0.782** | 0.759** | |  | | 2.519*** | | 2.536*** | |
|  |  | (0.441) | (0.384) |  | (0.297) | (0.280) | |  | | (0.528) | | (0.473) | |
| Unknown |  |  | -0.356 |  |  | -0.489 | |  | |  | | -1.406 | |
|  |  |  | (0.666) |  |  | (0.701) | |  | |  | | (1.615) | |
| **Subjective relative economic situation (Ref: both with middle or higher)** | | | | | | | | |  | |  | |  |
| Index with middle+, sib with low |  | -0.951** | -0.751** |  | 0.746** | 0.738** | |  | | 1.166* | | 1.077* | |
|  |  | (0.307) | (0.285) |  | (0.247) | (0.239) | |  | | (0.488) | | (0.458) | |
| Index with low, sib with middle+ |  | -0.738** | -0.740** |  | -0.861*** | -0.799** | |  | | -0.422 | | -0.409 | |
|  |  | (0.271) | (0.255) |  | (0.246) | (0.234) | |  | | (0.477) | | (0.431) | |
| Both with low |  | -1.727*** | -1.648*** |  | -0.103 | -0.156 | |  | | 1.044* | | 0.840† | |
|  |  | (0.355) | (0.335) |  | (0.281) | (0.271) | |  | | (0.499) | | (0.461) | |
| Unknown |  |  | -0.650 |  |  | -1.817* | |  | |  | | 1.181 | |
|  |  |  | (0.755) |  |  | (0.920) | |  | |  | | (1.098) | |
| **Parental vital status (Ref: No parents alive)** | | | |  |  |  | |  | |  | |  | |
| At least one parent alive |  | 0.646† | 0.620† |  | -0.263 | -0.371 | |  | | 1.146† | | 1.098† | |
|  |  | (0.371) | (0.361) |  | (0.339) | (0.339) | |  | | (0.608) | | (0.578) | |
| Unknown |  |  | -0.0638 |  |  | -0.387 | |  | |  | | 0.629 | |
|  |  |  | (0.407) |  |  | (0.362) | |  | |  | | (0.552) | |
| **Initial sibship size** |  | -0.225*** | -0.224*** |  | -0.125* | -0.129* | |  | | -0.0440 | | -0.058 | |
|  |  | (0.064) | (0.059) |  | (0.053) | (0.051) | |  | | (0.098) | | (0.089) | |
| **Intercepts (cumulative intercepts for meologit models)** | | | |  |  |  | |  | |  | |  | |
| Intercept 1 | 3.484*** | 12.079*** | 12.04*** | 3.608*** | 9.529*** | 9.360*** | |  | |  | |  | |
|  | (0.317) | (0.915) | (0.852) | (0.309) | (0.767) | (0.716) | |  | |  | |  | |
| Intercept 2 | 7.471*** | 17.194*** | 17.10*** | 6.425*** | 12.741*** | 12.62*** | |  | |  | |  | |
|  | (0.403) | (1.074) | (0.996) | (0.374) | (0.849) | (0.793) | |  | |  | |  | |
| **Constant (intercept for melogit models)** | | | |  |  |  | | -5.785*** | | -11.997^***^ | | -11.69*** | |
|  |  |  |  |  |  |  | | (0.594) | | (1.846) | | (1.594) | |
| **Log likelihood** | -1905.5552 | -1645.1504 | -1865.8807 | -1678.8551 | -1509.1132 | -1664.9137 | | -510.47267 | | -449.5264 | | -528.65276 | |
| **Wald chi2(11/31/36), Prob > chi2** | 155.68,  p < 0.001 | 293.31,  p < 0.001 | 327.10,  p < 0.001 | 127.90,  p < 0.001 | 236.46,  p < 0.001 | 267.40,  p < 0.001 | | 68.87,  p < 0.001 | | 62.07,  p < 0.001 | | 75.47,  p < 0.001 | |
| **Variance Level 2** | 9.508*** | 13.68*** | 13.43*** | 5.718*** | 6.328*** | 6.491*** | | 5.371*** | | 9.121** | | 8.718*** | |
|  | (1.248) | (1.991) | (1.825) | (0.865) | (1.002) | (0.958) | | (1.359) | | (3.066) | | (2.592) | |
| **Number of dyads** | 4,050 | 4,050 | 4,599 | 4,044 | 4,044 | 4,593 | | 4,038 | | 4,038 | | 4,587 | |
| **Number of index persons** | 1,699 | 1,699 | 1,925 | 1,698 | 1,698 | 1,923 | | 1,694 | | 1,694 | | 1,920 | |

*** *p* <0.001, ** *p* <0.01, * *p* <0.05, ^†^ *p* <0.1.

| **Frequency of receiving practical help** |  |
| --- | --- |
|  |  |
| **Frequency of giving practical help** |  |
|  |  |
| **Giving personal care** |  |
|  |  |

**Figure S1.** Average predicted probabilities from multilevel ordinal logistic regression results for core-kinlessness and the frequency of receiving practical help from siblings and giving it to siblings, as well as logistic regression results for the likelihood of giving personal care to siblings and their 95% confidence intervals

*Note*: All models control for the index person’s subjective health, language, sibling’s age, sibship size, having at least one parent alive, urbanity of index’s municipality, and such relative characteristics of the index person-sibling in a dyad as gender composition, type of sibling ties, relative subjective economic situation, and emotional closeness.

**Table S3.** Multilevel ordinal logistic regression results for the main explanatory variables and frequency of receiving practical help from siblings and giving it to siblings, as well as logistic regression results for the likelihood of giving personal care to siblings, coefficients, and 95% confidence intervals

|  | **Frequency of receiving practical help** | | | **Frequency of giving practical help** | | **Giving personal care** |
| --- | --- | --- | --- | --- | --- | --- |
| **Core-kinlessness status (Ref: both a partner and at least one child)** | | | | | | |
| No partner, child | 1.204*** | | | 0.947*** | | 1.188** |
|  | (0.284) | | | (0.242) | | (0.441) |
| Partner, no child | -0.076 | | | -0.045 | | -0.510 |
|  | (0.514) | | | (0.427) | | (0.955) |
| No partner, no child | 3.247*** | | | 1.398*** | | 1.280* |
|  | (0.427) | | | (0.337) | | (0.597) |
| **Sibling’s parental status (Ref: sibling has a child)** | | | | | | |
| Childless sibling | 0.230 | | | 1.331** | | 2.029*** |
|  | (0.215) | | | (0.197) | | (0.377) |
| **Distance between siblings (Ref: 11-20 km)** | | | | | | |
| 0–5 km | 1.313*** | | | 0.963** | | 1.100* |
|  | (0.325) | | | (0.300) | | (0.555) |
| 6–10 | 1.052** | | | 0.431 | | 0.418 |
|  | (0.376) | | | (0.342) | | (0.642) |
| 21–0 | -0.123 | | | -0.0397 | | -0.842 |
|  | (0.337) | | | (0.309) | | (0.626) |
| 51–100 | -0.291 | | | -0.430 | | -1.369* |
|  | (0.345) | | | (0.321) | | (0.675) |
| 101+ km | -0.947** | | | -0.835** | | -1.491** |
|  | (0.309) | | | (0.283) | | (0.565) |
| **Index’s subjective health (Ref: good)** | | | | | | |
| Fair | -0.077 | | | -0.125 | | 0.263 |
|  | (0.246) | | | (0.208) | | (0.391) |
| Poor | -0.606 | | | -1.051^†^ | | 0.337 |
|  | (0.628) | | | (0.547) | | (0.855) |
| **Index’s language (Ref: Finnish)** | |  | | |  | |
| Swedish | -0.519 | | | 0.099 | | -1.464 |
|  | (0.546) | | | (0.453) | | (1.191) |
| **Index’s urbanity of municipality (Ref: urban)** | | | | | | |
| Semi-urban municipality | 0.741* | | | 0.941*** | | 1.071* |
|  | (0.315) | | | (0.260) | | (0.470) |
| Rural municipality | 0.927** | | | 0.666* | | 0.727 |
|  | (0.342) | | | (0.290) | | (0.549) |
| **Gender composition (Ref: sister-sister)** | | |  | | | |
| Sister-brother | 0.266 | | | -0.680** | | -0.739* |
|  | (0.194) | | | (0.207) | | (0.374) |
| Brother-sister | 0.110 | | | 0.923*** | | -1.129* |
|  | (0.297) | | | (0.248) | | (0.542) |
| Brother-brother | 1.045*** | | | 1.250*** | | -0.318 |
|  | (0.291) | | | (0.254) | | (0.494) |
| **Sibling ties (Ref: full siblings)** | | | | | | |
| Same mother only | -0.007 | | | 0.207 | | 0.373 |
|  | (0.564) | | | (0.449) | | (0.795) |
| Same father only | -1.335 | | | -1.899^†^ | | -1.341 |
|  | (1.039) | | | (1.071) | | (1.785) |
| **Emotional closeness between siblings** | 2.310*** | | | 1.522*** | | 1.149*** |
|  | (0.164) | | | (0.132) | | (0.249) |
| **Sibling’s age (Ref: 66–75 years old or close to Index’s age)** | | | | | | |
| Sibling <60 years old | 0.572* | | | -0.424 | | -2.725** |
|  | (0.274) | | | (0.291) | | (0.993) |
| 61–65 | 0.248 | | | -0.117 | | -0.541 |
|  | (0.194) | | | (0.197) | | (0.439) |
| 76–80 | -0.792** | | | 0.100 | | 1.155** |
|  | (0.259) | | | (0.225) | | (0.430) |
| ≥81 years old | -1.981*** | | | 0.773** | | 2.499*** |
|  | (0.441) | | | (0.297) | | (0.526) |
| **Subjective relative economic situation (Ref: both with middle or higher)** | | | | | | |
| Index with middle+, sib with low | -0.968** | | | 0.744** | | 1.155* |
|  | (0.308) | | | (0.248) | | (0.488) |
| Index with low, sib with middle+ | -0.750** | | | -0.855** | | -0.378 |
|  | (0.271) | | | (0.246) | | (0.474) |
| Both with low | -1.730*** | | | -0.0788 | | 1.124* |
|  | (0.354) | | | (0.280) | | (0.500) |
| **Parental vital status (Ref: No parents alive)** | | | | | | |
| At least one parent alive | 0.604 | | | -0.274 | | 1.196* |
|  | (0.372) | | | (0.341) | | (0.608) |
| **Initial sibship size** | -0.217** | | | -0.122* | | -0.042 |
|  | (0.064) | | | (0.053) | | (0.098) |
| **Intercepts (cumulative intercepts for meologit models)** | | | | | | |
| Intercept 1 | 12.14*** | | | 9.476*** | |  |
|  | (0.914) | | | (0.763) | |  |
| Intercept 2 | 17.26*** | | | 12.69*** | |  |
|  | (1.074) | | | (0.846) | |  |
| **Constant (intercept for melogit models)** |  | | |  | | -11.70*** |
|  |  | | |  | | (1.804) |
| **Variance Level 2** | 13.88*** | | | 6.413*** | | 9.186*** |
|  | (2.027) | | | (1.013) | | (3.086) |
| **Number of dyads** | 4,050 | | | 4,044 | | 4,038 |
| **Number of index persons** | 1,699 | | | 1,698 | | 1,694 |

*** *p* <0.001, ** *p* <0.01, * *p* <0.05, ^†^ *p* <0.1.

***Interaction between siblings’ parenthood status and intragenerational geographic proximity***

We found that the index persons received practical help from childless siblings more frequently, irrespective of the distance, while the frequencies varied by distance if the sibling had children (Figure S2). Furthermore, our models showed that index persons provided childless siblings with help more frequently than siblings with children within the same distance categories, but they provided childless siblings living within 10 km with help more often than those living farther away. Similarly, index persons were most likely to give personal care to childless siblings living nearby than to childless siblings living more than 10 km away, then to close siblings with children, and, finally, to distant siblings with children.

| **Frequency of receiving practical help** |  |
| --- | --- |
|  |  |
| **Frequency of giving practical help** |  |
| **Sibling’s childlessness and proximity** |  |
|  |  |
| **Giving personal care** |  |
|  |  |

**Figure S2.** Multilevel ordinal logistic regression results for the variations in the effects of the geographic proximity on frequency of receiving practical help from siblings, giving it to siblings, and likelihood of giving personal care to siblings by the availability of siblings’ children, average predicted probabilities, and 95% confidence intervals

*Note*: All models control for the index person’s subjective health, language, sibling’s age, sibship size, having at least one parent alive, urbanity of index’s municipality, gender composition of a dyad, type of sibling ties, relative subjective economic situation, and emotional closeness.

**Figure S3.** Multilevel ordinal logistic regression results for the alternative categorization of the distance (used in SHARE) and frequency of receiving practical help from siblings and giving it to siblings as well as logistic regression results for the likelihood of giving personal care to siblings, coefficients, and 95% confidence intervals

*Note*: All models control for the index person’s subjective health, language, sibling’s age, sibship size, having at least one parent alive, urbanity of index’s municipality, gender composition of a dyad, type of sibling ties, relative subjective economic situation, and emotional closeness.
